# Transcriptome Analysis of the Chinese White Wax Scale *Ericerus pela* with Focus on Genes Involved in Wax Biosynthesis

**DOI:** 10.1371/journal.pone.0035719

**Published:** 2012-04-20

**Authors:** Pu Yang, Jia-Ying Zhu, Zhong-Jun Gong, Dong-Li Xu, Xiao-Ming Chen, Wei-Wei Liu, Xin-Da Lin, Yan-Fei Li

**Affiliations:** 1 Research Institute of Resources Insects, Chinese Academy of Forestry, Key Laboratory of Cultivating and Utilization of Resources Insects of State Forestry Administration, Kunming, China; 2 Key Laboratory of Forest Disaster Warning and Control of Yunnan Province, College of Forestry, Southwest Forestry University, Kunming, China; 3 Institute of Plant Protection, Henan Academy of Agricultural Science, Key Laboratory of Crop Pest Control of Henan Province, Zhengzhou, China; 4 College of Life Sciences, China Jiliang University, Hangzhou, China; Auburn University, United States of America

## Abstract

**Background:**

The Chinese white wax scale, *Ericerus pela* Chavannes is economically significant for its role in wax production. This insect has been bred in China for over a thousand years. The wax secreted by the male scale insect during the second-instar larval stage has been widespread used in wax candle production, wax printing, engraving, Chinese medicine, and more recently in the chemical, pharmaceutical, food, and cosmetics industries. However, little is known about the mechanisms responsible for white wax biosynthesis. The characterization of its larval transcriptome may promote better understanding of wax biosynthesis.

**Methodology/Principal Findings:**

In this study, characterization of the transcriptome of *E. pela* during peak wax secretion was performed using Illumina sequencing technology. Illumina sequencing produced 41,839 unigenes. These unigenes were annotated by blastx alignment against the NCBI Non-Redundant (NR), Swiss-Prot, KEGG, and COG databases. A total of 104 unigenes related to white wax biosynthesis were identified, and 15 of them were selected for quantitative real-time PCR analysis. We evaluated the variations in gene expression across different development stages, including egg, first/second instar larvae, male pupae, and male and female adults. Then we identified five genes involved in white wax biosynthesis. These genes were expressed most strongly during the second-instar larval stage of male *E. pela*.

**Conclusion/Significance:**

The transcriptome analysis of *E. pela* during peak wax secretion provided an overview of gene expression information at the transcriptional level and a resource for gene mining. Five genes related to white wax biosynthesis were identified.

## Introduction

The Chinese white wax scale (CWWS) (*Ericerus pela*), silk worm (*Bombyx mori*), and honey bee (*Apis cerana*) are the three insect species most famous for their role in economic production in China. Male CWWS are best known wax producers. CWWS have been bred in China for over a thousand years [1]. The CWWS are widely distributed in most parts of China and elsewhere, Japan, and the Korean peninsula from the subtropics to temperate regions [2]. They live mainly on Chinese privet (*Ligustrum lucidum*) and Chinese ash (*Fraxinus chinesis*). The male CWWSs secrete large amounts of pure white wax from wax glands. This wax is lustrous, free of pollutants, and has a high melting point and stable chemical properties. White wax has been used in candle production, printing, and traditional medicine for long periods of time. The application of white wax has expanded to the chemical, pharmaceutical, food, and cosmetic industries [3].

The components of white wax have been well defined. They consist of cerylcerotate as the major component and some even-numbered C26–C30 straight-chain, unsaturated fatty acids and their corresponding fatty alcohols, such as hexacosanol and octacosanol. There are also a few minor components [3]. Despite knowledge of the chemical components of white wax, no enzymes involved in the synthesis of white wax have been identified in the CWWS until the present study because most research efforts have been dedicated to the extraction, isolation, and purification of hexacosanol and octacosanol for their medicinal values.

Over the past decades, research on wax biosynthetic pathway has made remarkable progress. Many genes encoding enzymes that participate in the biosynthesis of wax have been functionally characterized in protozoa, plants, and animals [4]–[9]. The biosynthesis of very-long-chain fatty acids (VLCFAs) begins with C18 in the plastid, which is catalyzed by the long-chain fatty acid elongase (FAE) system. Fatty acid elongation occurs by cycling through a four-step reaction (condensation, reduction, dehydration, and reduction), with a two carbon extension of the fatty acid chain through each cycle [4]. It has been proposed that the condensation reaction is the rate-limiting step in the fatty acid elongation. The condensing enzymes are classified into two genetically distinct groups based on the results of comparative amino acid sequence analysis: the KCS (β-ketoacyl-CoA synthase)/FAE group in plants and the ELO (elongase) group in mammals and fungi [10], [11]. In the wax biosynthetic pathway, the fatty acyl CoA precursor was reduced to the corresponding alcohol by fatty acyl-CoA reductase (FAR) and proceeds through esterification with alcohol, which is catalyzed by the wax synthase (WS), yielding wax ester [4]–[8]. Enzymes that catalyzes the reduction of fatty acyl-CoAs to alcohols have been isolated from various organisms, including protozoa, plants, animals, and insects [5], [6], [12]–[21]. So far, only ten genes encoding WS have been functionally identified in mice (*Mus musculus*), *Gallus gallus domesticus*, *Anser anser domesticus*, *Tyto alba*, jojoba (*Simmondsia chinensis*), *Arabidopsis thaliana*, *Petunia hydrida*, *Euglena gracilis*, *Acinetobacter calcoaceticus*, and *Marinobacter hydrocarbonoclasticus* [7]–[9], [21]–[25].

**Figure 1:**
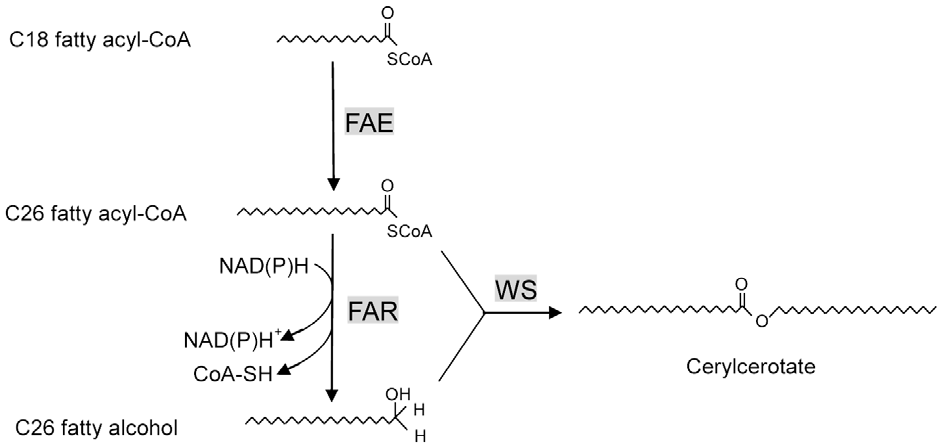
Biosynthesis of main component of white wax.

Cheng and Russell co-expressed the FAR and WS of mouse in human embryonic kidney (HEK) 293 cells, which resulted in the synthesis of wax monoesters [8]. Recently, Teerawanichpan et al. reconstituted the wax biosynthetic pathway by heterologous expression of FAR of *A. mellifera*, together with WS of *E. gracilis* in yeast (*Saccharomyces cerevisiae*) [13]. This led to high-level production of wax monoesters. Based on these findings, we believe that the main component of white wax of the CWWS is synthesized mainly by hexacosanol esterified with hexacosanoic acid, which is derived from C18 straight-chain fatty acids (Figure 1). The genes that participate in the white wax biosynthesis include FAE (mainly is ELO), FAR, WS, and ABC transporters, which transport wax to the surface.

CWWS is sexually dimorphic. The males undergo holometabolism and secrete wax continuously throughout the early and later stages of the second larval instar. The females undergo hemimetabolism and cannot produce white wax but are rather coated with hard chitin cuticles [26]. Yang et al. analyzed the soluble proteome of male CWWS cuticle at the second-instar larval stage and identified 278 protein groups (2584 peptides), but did not obtain useful information related to wax biosynthesis because there was no fully developed protein database for CWWS [26]. Although the biological characteristics of CWWS have been reported by many researchers, at present, there are no genomic resources available for CWWS. Progress in understanding the molecular mechanisms of wax biosynthesis and development of CWWS has been hindered by the dearth of information available for transcriptional, proteomic, and genetic functional analyses of CWWS.

We used Illumina technology in this study to provide an overview of the larva transcriptome of the CWWS during peak wax secretion. We expect that this will lead to a more complete understanding of the white wax biosynthetic pathway and its regulatory mechanism and facilitate gene mining for future physiological work on CWWS.

## Methods

### Insects

No specific permits were required for the described field studies.

The male CWWS during their second-instar larval stage, which is the peak time for wax secretion, were collected in the morning from one branch of *L. lucidum* in the Research Institute of Resources Insects. The bodies of CWWS were detached from the wax layers in the laboratory and homogenized in TRIZOL (Invitrogen, U.S.). Total RNA was extracted according to the manufacturer's protocol. RNA integrity was confirmed by the Agilent 2100 Bioanalyzer (Agilent Technologies) with clear characteristic peaks at 28S and 18S and an RNA integrity number (RIN).

### cDNA library preparation and Illumina sequencing

Twenty micrograms of total RNA was prepared for cDNA library construction according to the Illumina manufacturer's instructions. mRNA was isolated using magnetic oligo(dT) beads. Fragmentation buffer was added for interrupting mRNA to short fragments, and the short fragments were used as templates. Random hexamer-primer was used to synthesize first-strand cDNA. Buffer, dNTPs, RNase H, and DNA polymerase I were used to synthesize second-strand cDNA. After that, short fragments were purified using a QiaQuick PCR extraction kit and resolved with elution buffer for end reparation and the addition of poly(A). Then the short fragments were connected with sequencing adapters. The fragments were selected using the results of agarose gel electrophoresis, and suitable fragments were used as templates for PCR amplification. Finally, the library was sequenced using Illumina HiSeq 2000. The raw data has been deposited in SRA (NCBI).

### Reads assembly and sequence annotation

After filtering dirty raw reads, de novo assembly of transcriptome was carried out using a short read assembly program called SOAP [27]. Then blastx (BLAST, the basic local alignment search tool) alignment (E value<10^−5^) was performed between unigenes and protein databases, including NR (non-redundant database), Swiss-Prot, KEGG (Kyoto Encyclopedia of Genes and Genomes), and COG (cluster of orthologous groups). The best alignment results were used to determine the sequence direction of the unigenes. When the results of different databases conflicted with each other, they were ranked in the following order: NR, Swiss-Prot, KEGG, and COG. After alignment using these protein databases, we obtained protein and GO functional annotations for each unigene. We then used WEGO software to perform GO functional classification for all unigenes. KEGG annotation allowed us to find pathway annotation. Alignment to the COG database allowed us to predict and classify the possible functions of unigenes.

### Sequence alignment and Phylogenetic analysis

Amino acid sequences related to FAE, FAR, WS and ABC transporters were downloaded from NCBI, sequence alignments were conducted with the DNAMAN6.0 software. Amino acid sequences alignment was carried out using the software CLUSTALX1.83 before construction of phylogenetic trees, and the result was imported to software MEGA5.0. Phylogenetic trees were generated using the neighbour-joining method and bootstrapped with 1000 iterations to evaluate the branch strength of the tree [28], [29].

### BLAST search of the WS gene using bio-cloud computing

Considering the low homology among WS genes in bacteria, plants, and mammals, the annotation of WS genes in the transcriptome of CWWS was analyzed again using a bio-cloud computing program available online (https://cloud.genomics.cn/index.php/login/membersarea). Three sequences of WS genes from mice (AY611031 and AY611032) and humans (AY605053) were selected as reference sequences for nucleic acids and amino acid alignment separately with the coding sequences of CWWS. The results were filtered with a set of default parameters (E-value threshold: 1.00E-3; match/mismatch score: 1, −3; gap open/extend costs: 5, 2).

### Quantitative real-time PCR (qRT-PCR) analysis

Total RNA of CWWS at different development stages was extracted separately, and 2 µg total RNA of each sample was reverse-transcribed in a 20 µl reaction system according to the protocol provided with the M-MLV first strand kit (Invitrogen, China). A total of 15 genes were selected for qRT-PCR analysis. EvaGreen (Bio-RAD, U.S.) was used as DNA-binding fluorescent dye, and β-actin was used as an internal standard. The absolute concentrations of target genes at deferent developmental stages were determined using the standard curve quantitation method, and the statistical analyses were performed using the least significant difference (LSD) test at *P* = 0.01 and *P* = 0.05 with DPS statistical software [30].

## Results

### Illumina sequencing and sequence assembly

After cleaning the data and removing small reads, Illumina sequencing produced a total of 13,333,334 reads covering a total of 47,563,613 nt assembled into 353,779 contigs with an average size of 134 bp (ranging from 75 bp to 2065 bp). These contigs were assembled into 64,304 scaffolds with a mean length of 365 bp using paired end-joining and gap-filling. Paired-end reads were used again for gap filling of scaffolds to produce sequences with least Ns (N represents A/T/C/G) that could not be extended on either end. A total of 41,839 unigenes were generated with mean length of 482 bp, and 3,570 unigenes were over 1000 bp. The SRA accession number was SRA047286.1. To evaluate assembly accuracy, we selected 5 unigenes and designed 5 primer pairs for RT-PCR amplification. These primer pairs all yielded PCR products of the expected sizes whose sequences were further confirmed by Sanger sequencing (data not shown).

### Annotation of predicted proteins

**Figure 2:**
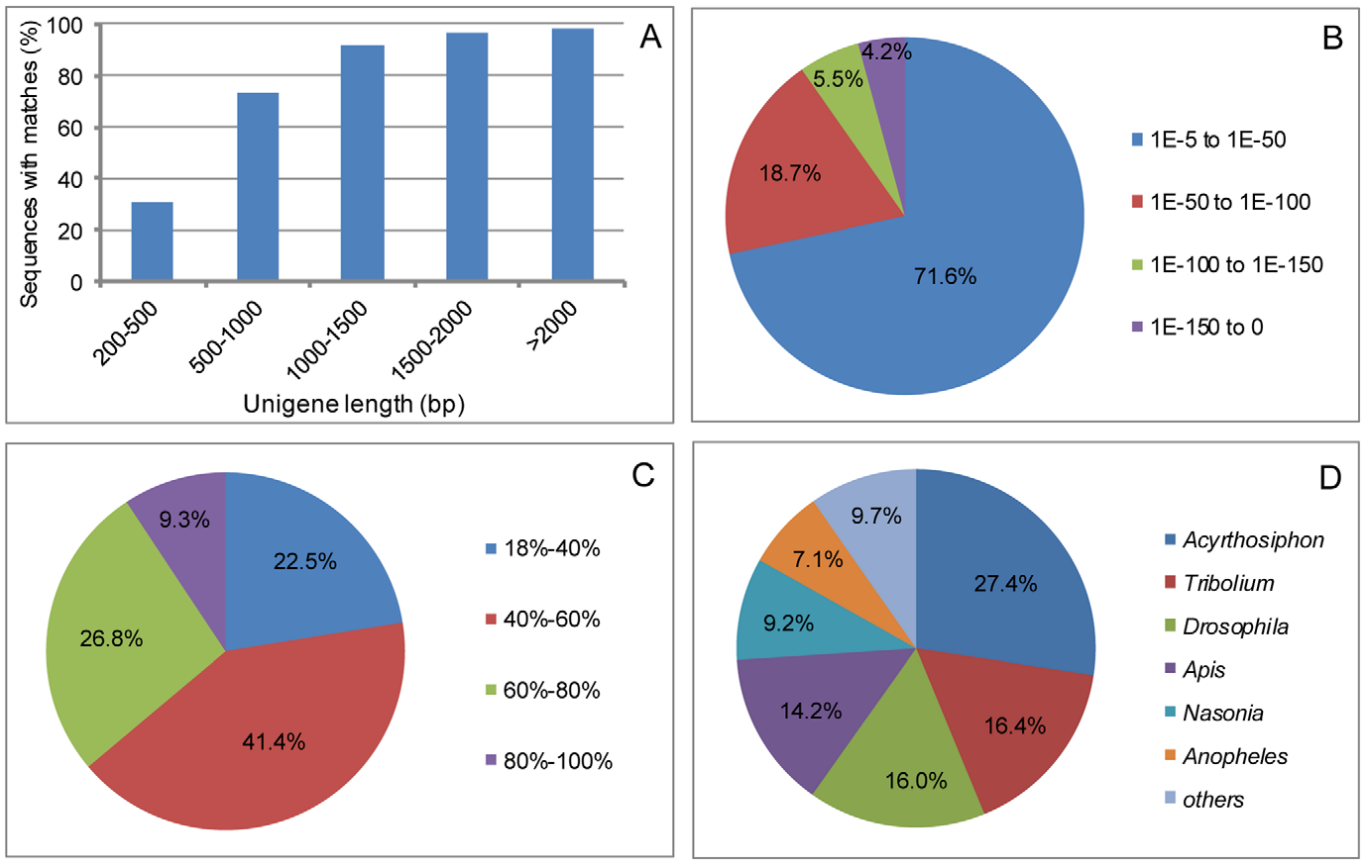
Analysis of Illumina sequences in the NR database. A. Proportion of sequences with matches; B. E-value distribution; C. Similarity distribution; D Species distribution.

To annotate these unigenes, blastx alignment (E value<10^−5^) against the NR, Swiss-Prot, KEGG, and COG databases was performed. A total of 18,861 unigenes (45.1% of all unigenes) returned above-cutoff BLAST results when searched against the NR nucleotide database, while the other 22,978 (54.9% of all unigenes) could not be matched to known genes. The statistical analysis of sequences in the NR database indicated that the proportion of sequences with matches was lower for the shorter sequences, especially sequences between 200 and 500 bp. Match efficiency above 90% was observed for sequences longer than 1,000 bp (Figure 2A). An E-value of 71.6% for the matched sequence ranged between 1.0E-5 to 1.0E-50 (Figure 2B). The similarity distribution showed that 41.4% sequences had a similarity ranging from 40% to 60%, while only 9.3% sequences had a similarity ranging from 80% to 100% (Figure 2C). The species distribution of the top hits in the NR database showed that 27.40% of the CWWS sequences matched with *Acyrthosiphon pisum*, followed by *Tribolium castaneum* (16.4%), *Drosophila* (16.0%), *A. mellifera* (14.2%), *Nasonia vitripennis* (9.2%), and *Anopheles gambiae* (7.1%) (Figure 2D). In addition, 15,406 unigenes were matched to proteins in Swiss-Prot.

**Figure 3:**
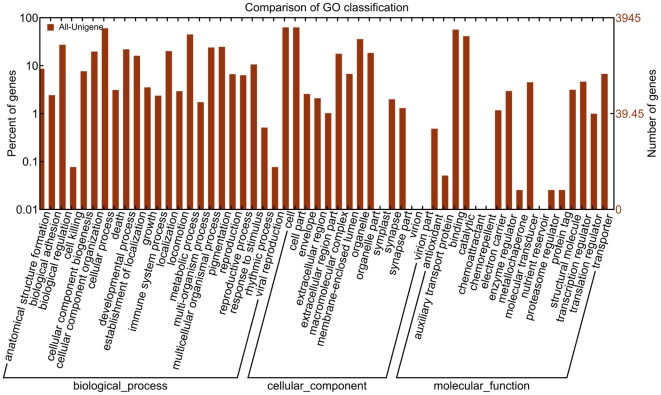
GO categories of the unigenes. Unigenes were annotated in three main categories: biological process, cellular component and molecular function.

All the predicted CWWS unigenes were subjected to gene ontology analysis to categorize the list of genes based on biological processes, cellular components, and molecular function. Of the 41,839 unigenes, 3,945 were assigned specific GO terms and categorized into 23 biological processes, 14 cellular components, and 17 molecular functions. “Cellular component organization”, “viral reproduction”, “cell”, and “auxiliary transport protein” terms were dominant (Figure 3).

**Figure 4:**
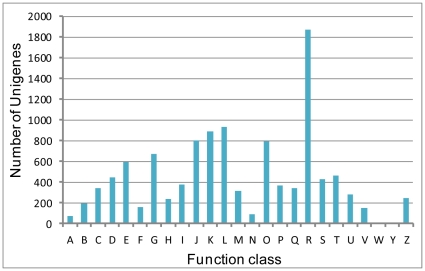
COG classification of the unigenes. A: RNA processing and modification; B: Chromatin structure and dynamics; C: Energy production and conversion; D: Cell cycle control, cell division, chromosome partitioning; E: Amino acid transport and metabolism; F: Nucleotide transport and metabolism; G: Carbohydrate transport and metabolism; H: Coenzyme transport and metabolism; I: Lipid transport and metabolism; J: Translation, ribosomal structure and biogenesis; K: Transcription; L: Replication, recombination and repair; M: Cell wall/membrane/envelope biogenesis; N: Cell motility; O: Posttranslational modification, protein turnover, chaperones; P: Inorganic ion transport and metabolism; Q: Secondary metabolites biosynthesis, transport and catabolism; R: General function prediction only; S: Function unknown; T: Signal transduction mechanisms; U: Intracellular trafficking, secretion, and vesicular transport; V: Defense mechanisms; W: Extracellular structures; Y: Nuclear structure; Z: Cytoskeleton.

COG classifications were also used to classify the functions of the CWWS unigenes. Of the unigenes examined, 6,245 were classified as COG. The largest set of unigenes was assigned to the cluster of “general function prediction” (1873, 16.96%), followed by “replication, recombination, and repair” (933, 8.45%), “transcription” (885, 8.01%). The categories of “extracellular structures” (2, 0.02%) and “nuclear structure” (7, 0.06%) represent the smallest groups (Figure 4). The other 20 categories are shown in Figure 4.

All identified sequences were also subjected to KEGG to identify the biological pathways most active in the CWWS. Of these, 12,153 unigenes were annotated to KEGG. In total, 203 pathways were obtained including “peroxisome” (261 members), “ABC transporters” (150 members) and “fatty acid biosynthesis” (91 members), which involved in the wax synthesis and transportation.

### Detections of sequences related to the wax biosynthesis

In the process of wax synthesis, fatty alcohols are esterified with fatty acyl-CoA, which is catalyzed by WS. We analyzed the genes related to elongation of fatty acids, fatty alcohol synthesis, wax synthesis, and transportation. A total of 104 unigenes related to white wax biosynthesis were identified. As shown in Table S1, we identified 6 sequences whose top hits were ELO in the NR and Swiss-Prot databases. In total, we obtained 43 sequences whose top hits were FAR genes in the Swiss-Prot and KEGG databases (Table S2).

**Figure 5:**
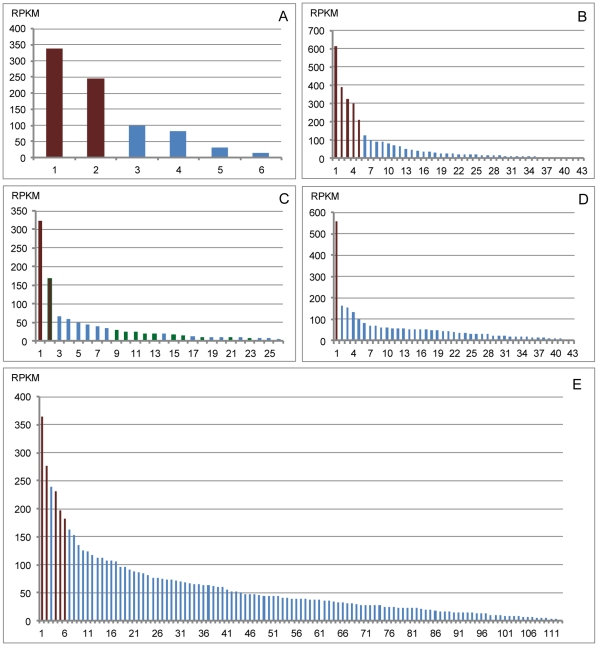
RPKM of putative ELO, FAR and WS genes. A, RPKM of 6 ELO genes; B, RPKM of 43 FAR genes; C, RPKM of 26 enzymes of acyltransferase family, green bars represent the WS genes; D, RPKM of 44 ABC transporters genes; E, RPKM of 113 putative WS genes through BLAST search using bio-cloud computing. Red bars of each histogram represent the genes with the highest RPKM and are selected for qRT-PCR analysis.

WS is a member of the acyltransferase family, which includes diacylglycerol acyltransferase (DGAT), monoacylglycerol acyltransferase (MGAT), and acylCoA: cholesterol acyltransferase (ACAT). We obtained 26 sequences similar to the acyltransferase family in the NR database, and 11 of them showed at least one hit with WS genes (Table S3, Figure 5C). However, none of them were the top hit in the protein database. We also obtained 10 sequences in the KEGG database showed at least one hit with WS genes, however their unigene ID repeated the above. We obtained no WS genes in other databases, including Swiss-Prot, GO, and COG. Bio-cloud computing results showed that 113 sequences shared homology with sequences of WS genes of mice and humans (Figure 5E). In addition, 44 ABC transporter genes were detected in the CWWS transcriptome (Table S4).

### Sequence alignment and Phylogenetic analysis

The sequence alignments and phylogenetic analysis results showed that, the ELO, FAR, WS, and ABC transporters genes of *E. pela* share high identity with the homologues from other species, especially with that of insects (Figure S1, S2, S3, S4, S5, S6, S7, S8). The conserved Histidine box was found in the ELO of *E. pela* (Figure S1). The characteristic domains of eukaryotic FARs, including a NADH-binding motif, a Rossmann-fold NAD(P)(+)-binding domain, and a Sterile domain were found in the amino acid sequence of *E. pela* (Figure S2) according to Liénard et al (2010). In the phylogenetic analyses, WS of *E. pela* was remote from the birds WS clade but clustered near the DGAT2 clade (Figure S7). The conserved motif HHXXXDG of WSs in the *P. hydrida* and *A. calcoaceticus* was not found in *E. pela* and animals (Figure S3).

### qRT-PCR analysis

**Figure 6:**
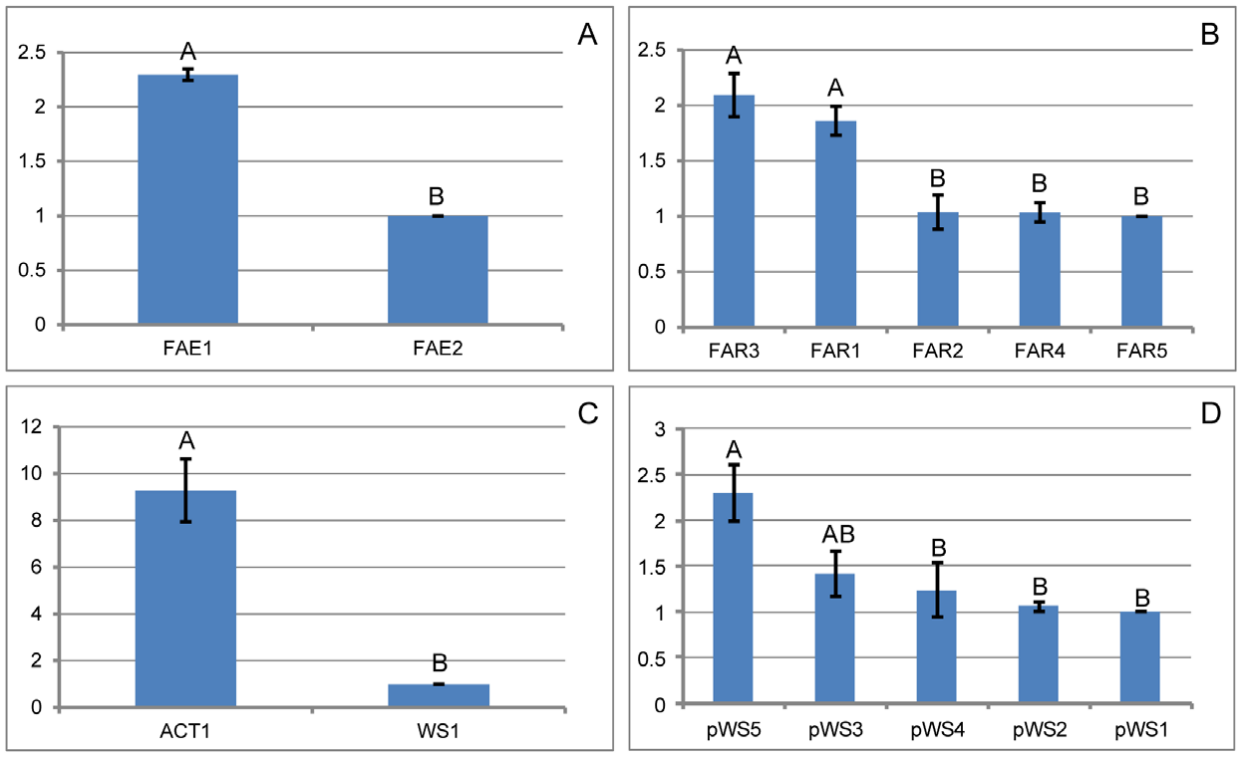
Relative expression levels of the candidate genes related to wax biosynthesis at the second-instar larval stage. A, relative quantification analysis of two ELO genes; B, relative quantification analysis of five FAR genes; C, relative quantification analysis of one sequence of acyltransferase (ACT1) and one WS gene (WS1); D, relative quantification analysis of five putative WS genes (Pws1-5) through BLAST search using bio-cloud computing. The same letters are not significantly different, *P*>0.01.

**Figure 7:**
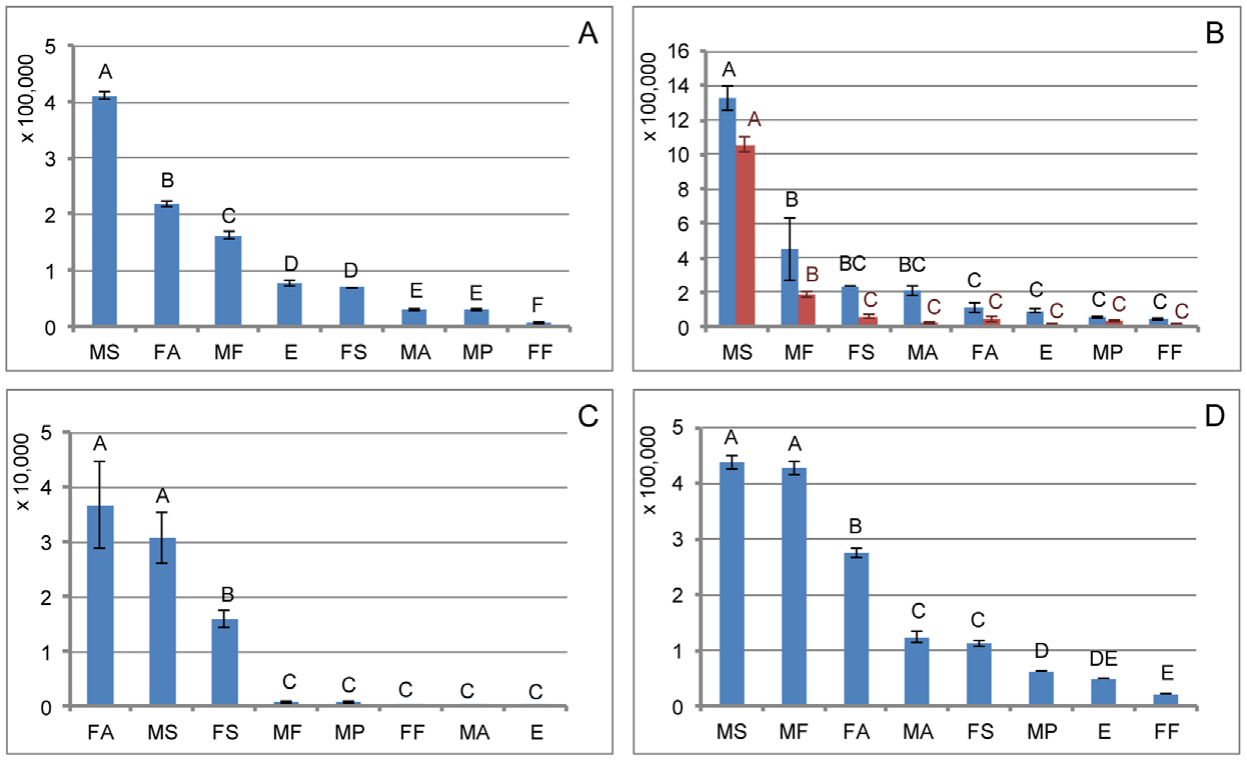
Absolute quantification analysis of the genes involved in wax biosynthesis during different developmental stages. A, Absolute quantification analysis of ELO1; B, Absolute quantification analysis of FAR3 (blue bars) and FAR1 (red bars); C, Absolute quantification analysis of WS1; D, Absolute quantification analysis of ABC1. The same letters are not significantly different, *P*>0.01. MS: male second-instar larvae, the peak time of wax secretion; MF: male first-instar larvae; MP: male pupae; MA: male adults; FF: female first-instar larvae; FS: female second-instar larvae; FA: female adults; E: eggs.

The genes with the highest transcript abundance were thought to be involved in wax biosynthesis, their relative expression levels were determined by RPKM (reads per kb per million reads) and qRT-PCR analysis (the primers used for qRT-PCR are shown in Table S5). Two of the six ELO genes, ELO1 and ELO2, exhibited higher RPKM value than the others (Figure 5A) and were selected for qRT-PCR analysis. The relative quantification analysis showed that the ELO1 gene was expressed at a significantly higher level than ELO2 (Figure 6A). It was selected for absolute quantification analysis across different development stages. The results showed that the ELO1 gene was most highly expressed in male second-instar larvae, and the second instar is the peak time of wax secretion (Figure 7A). This gene is thought to be involved in the elongation of fatty acids during white wax biosynthesis.

Five FAR genes (FAR1–5) demonstrated higher RPKM values than the others (Figure 5B). FAR1 and FAR3 were expressed significantly more highly when compared with the other three (Figure 6B). Therefore, we focused our efforts on the evaluation of the relative expression of the two FAR genes during different developmental stages. The absolute quantification analysis result showed that the two genes involved in the fatty alcohol synthesis expressed highest in male second-instar larvae (Figure 7B).

**Figure 8:**
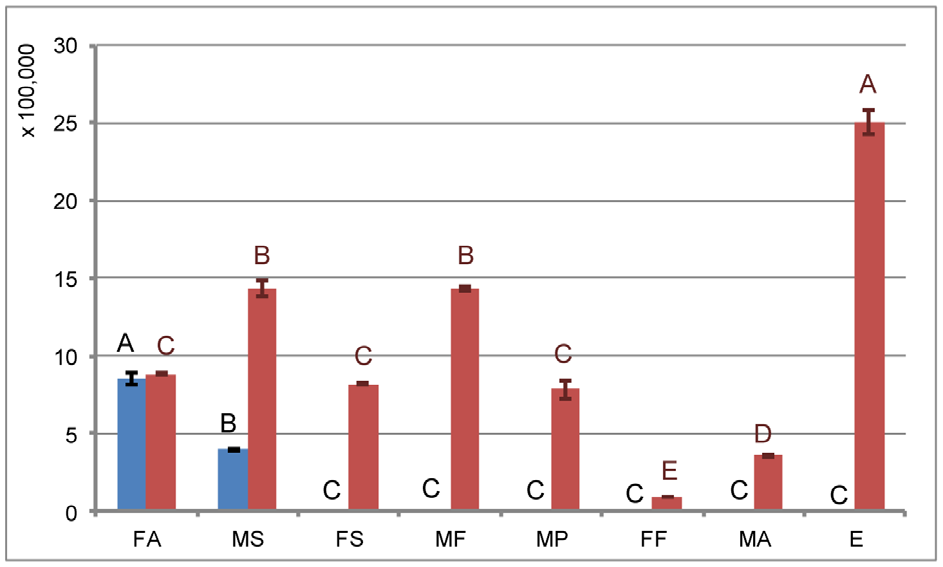
Absolute quantification analysis of ACT1 (blue bars) and pWS5 (red bars) during different developmental stages. The same letters are not significantly different, *P*>0.01.

One WS gene (WS1) and one acyltransferase sequence (ACT1) with the highest RPKM value were selected for mRNA relative expression analysis (Figures 5C and 6C). The two sequences were further analyzed by mRNA absolute expression to determine variations in gene expression during different developmental stages in male and females CWWS (Figure 7C and Figure 8). The results showed that the WS1 gene was most highly expressed in male second-instar larvae and adult females, while the ACT1 sequence was most highly expressed in female adults.

According to the results of bio-cloud computing, 8 of the 113 sequences have higher RPKM values than the others (Figure 5E). However, 3 of these 8 are similar to known genes, such as chitin deacetylase and titins. Only 5 sequences (putative WS genes, pWS1-5) were selected for relative quantification analysis (Figure 5E and Figure 6D). The results showed that pWS5 exhibited significantly higher expression than the others (*P*<0.05). It was therefore selected for mRNA absolute expression analysis. The results showed that it was most highly expressed during the egg stage, in which no white wax is secreted (Figure 8).

We identified 44 ABC transporters sequences. One sequence (ABC1) had the highest RPKM value when compared with the others (Figure 5D). So, it was selected for mRNA absolute expression analysis. The results showed that it expressed highest at the second and first instar larvae of males (Figure 7D).

## Discussion

The CWWS has been bred in China for commercial wax production for over a thousand years [3]. Although the ecological and biological characteristics of CWWS have been widely studied by many researchers, the mechanisms underlying wax biosynthesis have remained unclear. Research on the mechanism of white wax biosynthesis has important significance for white wax production. In this study, we analyzed the transcriptome of the CWWS at the early stage of the second-instar larva. A total of 41,839 unigenes were generated and 45.1% of them returned above-cutoff BLAST results. These results showed that the CWWS most similar to *A. pisum* when its transcriptome was compared to that of other insect species. This may be because they all belong to Hemiptera and are closely related evolutionarily. Upon examination 3,945 genes could be annotated to GO, 6,245 to COG, and 12,153 to KEEG. The annotated transcriptome sequences provide an invaluable resource for the identification of genes related to wax biosynthesis and transport.

It appears that the condensing reaction acts as a rate-limiting step that confers the chain-length specificity of each fatty acid elongation reaction [10], [11]. Therefore, we focused our efforts on the genes encoding the condensing enzymes ELO. By performing a deep sequencing analysis of the database, we found an ELO that was most highly expressed during the second-instar larval stage, which is the peak time for wax secretion. This may be the enzyme that catalyzes octadecanoic acid to form hexacosanolic acid.

Fatty alcohols are not only the precursors of wax biosynthesis, but also components of insect cuticular hydrocarbons and sexual pheromones [16]–[20], [31]. So far, only several FAR genes from insects have been functionally identified. Most of them are from pheromone glands of *B. mori*, *Ostrinia scapulalis*, *O. nubilalis*, *Heliothis virescens*, and *Yponomeuta* [16]–[20]. These glands are involved in the pheromone biosynthesis. Recently a FAR gene assumed to be involved in either pheromone or ether lipid biosynthesis was isolated from the head of *A. mellifera* [13]. However, no FAR gene responsible for wax biosynthesis in insects was identified. The *A. thaliana* genome contains eight FAR genes homologous to those of the jojoba, silk moth, and mouse [32]. In this transcriptome analysis, we identified 43 FAR genes. Many of the partial gene sequences obtained may belong to the same gene. The high abundance of FAR transcripts implies that they may be involved in white wax biosynthesis, pheromone biosynthesis, or ether lipid biosynthesis. Most of the 43 sequences were not expected to be involved in white wax biosynthesis. At the peak time of wax secretion of CWWS, the high transcriptional activity of the FAR genes involved in white wax biosynthesis provides a large number of reads and relatively high expression. Therefore, the FAR sequences with the highest RPKM values are considered likely to be FAR genes involved in white wax biosynthesis. Additional priority was given to FAR genes with significantly lower expression at other developmental stages in male and female CWWS. We identified 2 FAR genes involved in white wax biosynthesis.

WS sequences indicated that it is a member of the acyltransferase family of enzymes involved in the esterification of acyl acceptors, including fatty alcohols, 1, 2 and 1, 3-diacylglycerols, monoacylglycerol, α, ω-diols, and even thiols [8], [22]. The acyltransferase enzymes include MGAT, which produce diacylglycerols; DGAT, which produce triacylglycerols; and ACAT, which produce cholesteryl esters. In mice, the WS had little activity to synthesize diacylglycerols and triacylglycerols, but other acyltransferases, such as MGAT and ACAT, exhibited modest wax monoester synthesis activities [8]. But in *G. g. domesticus*, *A. a. domesticus*, *T. alba*, *A. thaliana*, and *A. calcoaceticus*, WS is a bifunctional enzyme (wax ester synthase/Acyl-CoA: diacylglycerol acyltransferase), which mediates not only wax ester but also triacylglycerol biosynthesis [9], [23], [24]. Up to date, no WS gene has been identified in insects. Based on the NR annotation, we obtained 11 sequences with at least one hit with WS genes. Considering the sequence similarity among acyltransferase enzymes, we identified another 15 sequences that were similar to MGAT, DGAT, or ACAT. In addition, we found 113 sequences that share homology with WS genes in mice and humans. A total of 7 candidate WS genes were selected for mRNA expression analysis by qRT-PCR. One of them was expressed at significantly higher levels during peak wax secretion than during other developmental stages. This enzyme may be responsible for white wax biosynthesis. The increased expression of this enzyme in adult females suggests that it may also be involved in biosynthesis of triacylglycerols, which are the dominating storage lipid in animals, plants, and eukaryotic microorganisms [24]. The ability of the WS enzyme to produce triacylglycerols or diacylglycerols in CWWS needs further studies.

After biosynthesis along the pathway, white wax must be transported to the surface. It has been proposed that the ABC transporters play a role in this process [4], [33]. Therefore, we analyzed changes in the expression of ABC transporters during different development stages. The results showed that ABC1 was most highly expressed during the first and second instars of male larvae, indicating that it is likely to be involved in wax transport.

The present transcriptome analysis provides valuable information for understanding the process of wax biosynthesis. Identifying the subcellular and histological locations of wax-synthesis-related genes and determining enzymatic activities will further disclose the mechanism of wax secretion of CWWS. These results are of considerable importance to other studies and may also have important practical applications.

## Supporting Information

Table S1 **ELO genes identified in the protein database.**
(DOC)

Table S2 **FAR genes identified in the protein database.**
(DOC)

Table S3 **Enzymes of acyltransferase family identified in NR database.**
(DOC)

Table S4 **ABC transporter genes identified in the protein database.**
(DOC)

Table S5 **Primers used for qRT-PCR.**
(DOC)

Figure S1 **Alignment of the deduced amino acid sequences of ELO.** Identical amino acid residues and conservative substitutions are shaded in black or gray respectively. The conserved Histidine box is underlined. The GenBank accession numbers of the sequences are as follows: *Drosophila melanogaster* NM_141699.3, *Bactrocera dorsalis* HQ148712.1, *Glossina morsitans morsitans* ADD19917.1, *Heliothis virescens* ACX53823.1, *H. sapiens* NP_060240, *M. musculus* NP_062296, *Rattus norvegicus* NP_599209, *Saccharomyces cerevisiae* NP_012339, *Caenorhabditis elegans* AF244356.
(TIF)

Figure S2 **Alignment of the deduced amino acid sequences of FAR.** The Rossmann-fold domain is shown in black box, the NADH-binding motif is double underlined, and the Sterile protein domain is underlined. The GenBank accession numbers of the sequences are as follows: *O. nubilalis* FJ807735, *B. mori* BAC79426, *H. sapiens* AAT42129, *A. thaliana* NP567936.
(TIF)

Figure S3 **Alignment of the deduced amino acid sequences of WS.** The GenBank accession numbers of the sequences are as follows: *M. musculus* AY611032, *H. sapiens* AY605053, *G. gallus* WS1 XP_424082.2, *G. gallus* WS4 XP_419207.1, *G. gallus* WS5 NP_001026192.1, *A. domesticus* WS5 Q031647, *T. alba* WS5 JQ031646, *A. domesticus* WS4 JQ031643, *T. alba* WS4 JQ031645.
(TIF)

Figure S4 **Alignment of the deduced amino acid sequences of ABC transporters.** The GenBank accession numbers of the sequences are as follows: *Nasonia vitripennis* XP_003426604.1, *Acyrthosiphon pisum* XP_001945365.2, *Harpegnathos saltator* EFN84917.1, *Bombus terrestris* XP_003401420.1, *Apis mellifera* XP_393164.4, *Acromyrmex echinatior* EGI67545.1, *Harpegnathos saltator* EFN78194.1, *Aedes aegypti* XP_001650952.1, *Culex quinquefasciatus* XP_001862847.1, *Daphnia pulex* EFX71377.1, *Camponotus floridanus* EFN69284.1, *Oreochromis niloticus* XP_003459375.1, *Tribolium castaneum* XP_973444.1, *Harpegnathos saltator* EFN84918.1, *Danio rerio* XP_687003.3.
(TIF)

Figure S5 **Phylogenetic tree of ELOs.**
(TIF)

Figure S6 **Phylogenetic tree of FARs.**
(TIF)

Figure S7 **Phylogenetic tree of WSs, DGATs, MOGATs, and ACATs.**
(TIF)

Figure S8 **Phylogenetic tree of ABC transporters.**
(TIF)
