# Supplementary material for: Transcriptome Analysis of the Chinese White Wax Scale Ericerus pela with Focus on Genes Involved in Wax Biosynthesis
Source: PLoS One. 2012 Apr 20;7(4):e35719. doi: 10.1371/journal.pone.0035719 (PMC3334986; doi:10.1371/journal.pone.0035719)
Supplement: Table S1 — ELO genes identified in the protein database. (DOC) [file pone.0035719.s001.doc]

**Table S1** ELO genes identified in the protein database.

| Unigene ID | Length | Protein database | Subject ID | Species | E value |
| --- | --- | --- | --- | --- | --- |
| Unigene24614 | 261 | NR | ADD19917.1 | *Glossina morsitans morsitans* | 1.00E-34 |
| Unigene27053 | 289 | Swissprot | P49191 | *Caenorhabditis elegans* | 1.00E-15 |
| Unigene35455 | 529 | Swissprot | Q6PC64 | *Danio rerio* | 5.00E-13 |
| Unigene36642 | 602 | Swissprot | A1L3X0 | *Homo sapiens* | 4.00E-10 |
| Unigene37446 | 665 | Swissprot | Q5ZJR8 | *Gallus gallus* | 1.00E-49 |
| Unigene10466 | 448 | Swissprot | Q6PC64 | *Danio rerio* | 7.00E-08 |
