# Supplementary material for: Transcriptome Analysis of the Chinese White Wax Scale Ericerus pela with Focus on Genes Involved in Wax Biosynthesis
Source: PLoS One. 2012 Apr 20;7(4):e35719. doi: 10.1371/journal.pone.0035719 (PMC3334986; doi:10.1371/journal.pone.0035719)
Supplement: Table S2 — FAR genes identified in the protein database. (DOC) [file pone.0035719.s002.doc]

**Table S2** FAR genes identified in the protein database.

| Unigene ID | Length | Protein database | Subject ID | Species | E value |
| --- | --- | --- | --- | --- | --- |
| Unigene10528 | 375 | Swissprot | A1ZAI5 | *Drosophila melanogaster* | 3.00E-18 |
| Unigene12790 | 532 | Swissprot | Q7ZXF5 | *Xenopus laevis* | 4.00E-39 |
| Unigene12962 | 894 | Swissprot | Q66H50 | *Rattus norvegicus* | 4.00E-56 |
| Unigene13102 | 461 | Swissprot | Q7ZXF5 | *Xenopus laevis* | 4.00E-36 |
| Unigene15304 | 1007 | Swissprot | A1ZAI5 | *Drosophila melanogaster* | 1.00E-51 |
| Unigene16322 | 201 | Swissprot | Q7ZXF5 | *Xenopus laevis* | 5.00E-09 |
| Unigene16348 | 201 | Swissprot | Q5ZM72 | *Gallus gallus* | 7.00E-15 |
| Unigene17770 | 210 | Swissprot | A1ZAI5 | *Drosophila melanogaster* | 9.00E-06 |
| Unigene21413 | 236 | Swissprot | A1ZAI5 | *Drosophila melanogaster* | 1.00E-11 |
| Unigene22594 | 245 | KEGG | K13356 |  | 6.00E-11 |
| Unigene22812 | 246 | Swissprot | A1ZAI5 | *Drosophila melanogaster* | 2.00E-14 |
| Unigene22846 | 246 | KEGG | K13356 |  | 3.00E-07 |
| Unigene23817 | 254 | Swissprot | A1ZAI5 | *Drosophila melanogaster* | 1.00E-06 |
| Unigene23 | 457 | Swissprot | Q7ZXF5 | *Xenopus laevis* | 3.00E-15 |
| Unigene24236 | 258 | Swissprot | A1ZAI5 | *Drosophila melanogaster* | 3.00E-06 |
| Unigene24274 | 258 | KEGG | K13356 |  | 3.00E-08 |
| Unigene24348 | 259 | Swissprot | A1ZAI5 | *Drosophila melanogaster* | 4.00E-12 |
| Unigene25530 | 271 | Swissprot | A1ZAI5 | *Drosophila melanogaster* | 1.00E-06 |
| Unigene26420 | 281 | Swissprot | A1ZAI5 | *Drosophila melanogaster* | 1.00E-12 |
| Unigene2720 | 303 | Swissprot | A1ZAI5 | *Drosophila melanogaster* | 2.00E-15 |
| Unigene28267 | 308 | Swissprot | Q7ZXF5 | *Xenopus laevis* | 3.00E-27 |
| Unigene288 | 1235 | Swissprot | A1ZAI5 | *Drosophila melanogaster* | 1.00E-91 |
| Unigene29486 | 330 | Swissprot | A1ZAI5 | *Drosophila melanogaster* | 4.00E-17 |
| Unigene29557 | 332 | Swissprot | A1ZAI5 | *Drosophila melanogaster* | 2.00E-21 |
| Unigene30001 | 341 | Swissprot | A1ZAI5 | *Drosophila melanogaster* | 2.00E-08 |
| Unigene31005 | 365 | Swissprot | Q7ZXF5 | *Xenopus laevis* | 1.00E-22 |
| Unigene32011 | 393 | Swissprot | A1ZAI5 | *Drosophila melanogaster* | 3.00E-13 |
| Unigene32204 | 398 | Swissprot | Q960W6 | *Drosophila melanogaster* | 3.00E-17 |
| Unigene3235 | 332 | Swissprot | Q7ZXF5 | *Xenopus laevis* | 1.00E-08 |
| Unigene33015 | 424 | Swissprot | A1ZAI5 | *Drosophila melanogaster* | 3.00E-30 |
| Unigene35244 | 517 | Swissprot | A1ZAI5 | *Drosophila melanogaster* | 2.00E-21 |
| Unigene35735 | 545 | Swissprot | A1ZAI5 | *Drosophila melanogaster* | 6.00E-35 |
| Unigene37255 | 650 | Swissprot | A1ZAI5 | *Drosophila melanogaster* | 9.00E-39 |
| Unigene38139 | 729 | Swissprot | A1ZAI5 | *Drosophila melanogaster* | 3.00E-57 |
| Unigene38431 | 763 | Swissprot | A1ZAI5 | *Drosophila melanogaster* | 5.00E-30 |
| Unigene39091 | 847 | Swissprot | A1ZAI5 | *Drosophila melanogaster* | 5.00E-28 |
| Unigene40587 | 1175 | Swissprot | Q960W6 | *Drosophila melanogaster* | 1.00E-116 |
| Unigene5025 | 552 | Swissprot | A1ZAI5 | *Drosophila melanogaster* | 7.00E-22 |
| Unigene6514 | 314 | Swissprot | Q7ZXF5 | *Xenopus laevis* | 3.00E-08 |
| Unigene6580 | 212 | Swissprot | A1ZAI5 | *Xenopus laevis* | 8.00E-06 |
| Unigene8111 | 1167 | Swissprot | Q922J9 | *Mus musculus* | 7.00E-66 |
| Unigene9510 | 260 | Swissprot | Q960W6 | *Drosophila melanogaster* | 1.00E-09 |
| Unigene9809 | 679 | Swissprot | A1ZAI5 | *Drosophila melanogaster* | 2.00E-10 |
