# Supplementary material for: Transcriptome Analysis of the Chinese White Wax Scale Ericerus pela with Focus on Genes Involved in Wax Biosynthesis
Source: PLoS One. 2012 Apr 20;7(4):e35719. doi: 10.1371/journal.pone.0035719 (PMC3334986; doi:10.1371/journal.pone.0035719)
Supplement: Table S3 — Enzymes of acyltransferase family identified in NR database. (DOC) [file pone.0035719.s003.doc]

**Table S3** Enzymes of acyltransferase family identified in NR database

| Unigene ID | Length | Annotated protein | Subject ID | Species | E value |
| --- | --- | --- | --- | --- | --- |
| Unigene14759 | 821 | WS1 | NP_001102841.1 | *Rattus norvegicus* | 1.00E-37 |
|  |  | MGAT2 | XP_975146.1 | *Tribolium castaneum* | 5.00E-39 |
|  |  | DGAT2 | XP_549058.2 | *Canis familiaris* | 1.00E-39 |
| Unigene18038 | 212 | WS1 | NP_001102841.1 | *Rattus norvegicus* | 7.00E-06 |
|  |  | DGAT2 | XP_001925128.1 | *Sus scrofa* | 1.00E-07 |
| Unigene22100 | 241 | WS2 | XP_001069359.1 | *Rattus norvegicus* | 4.00E-10 |
|  |  | MGAT1 | XP_975155.1 | *Tribolium castaneum* | 3.00E-11 |
|  |  | DGAT2 | XP_001925128.1 | *Sus scrofa* | 4.00E-14 |
| Unigene24613 | 261 | WS1 | XP_002720118.1 | *Oryctolagus cuniculus* | 1.00E-12 |
|  |  | MGAT3 | XP_002722878.1 | *Oryctolagus cuniculus* | 2.00E-14 |
| Unigene29558 | 332 | WS1 | XP_002720118.1 | *Oryctolagus cuniculus* | 2.00E-24 |
|  |  | MGAT3 | XP_001371248.1 | *Monodelphis domestica* | 8.00E-27 |
|  |  | DGAT2 | NP_001025367.1 | *Danio rerio* | 1.00E-23 |
| Unigene31365 | 375 | WS2 | XP_002720111.1 | *Oryctolagus cuniculus* | 7.00E-09 |
|  |  | MGAT2 | XP_393396.2 | *Apis mellifera* | 6.00E-13 |
|  |  | DGAT2 | XP_001653515.1 | *Aedes aegypti* | 2.00E-11 |
| Unigene32539 | 408 | WS1 | XP_002720118.1 | *Oryctolagus cuniculu* | 2.00E-10 |
|  |  | MGAT3 | XP_875499.2 | *Bos taurus* | 2.00E-11 |
| Unigene39282 | 876 | WS1 | XP_002720118.1 | *Oryctolagus cuniculus* | 3.00E-25 |
|  |  | MGAT3 | XP_850305.1 | *Canis familiaris* | 4.00E-27 |
|  |  | DGAT2 | XP_001925128.1 | *Sus scrofa* | 2.00E-25 |
| Unigene39622 | 932 | WS1 | NP_001102841.1 | *Rattus norvegicus* | 9.00E-47 |
|  |  | MGAT2 | NP_001092606.1 | *Bos taurus* | 1.00E-49 |
|  |  | DGAT2 | EFA83646.1 | *Polysphondylium pallidum* | 4.00E-49 |
| Unigene7000 | 270 | WS1 | NP_001102841.1 | *Rattus norvegicus* | 2.00E-08 |
|  |  | MGAT2 | XP_001815791.1 | *Tribolium castaneum* | 7.00E-09 |
|  |  | DGAT2 | XP_001490162.1 | *Equus caballus* | 2.00E-09 |
| Unigene8905 | 726 | WS1 | XP_002720118.1 | *Oryctolagus cuniculus* | 5.00E-22 |
|  |  | MGAT3 | XP_875499.2 | *Bos taurus* | 7.00E-23 |
|  |  | DGAT2 | AAK84178.1 | *Homo sapiens* | 1.00E-21 |
| Unigene34475 | 482 | MGAT2 | XP_393396.2 | *Apis mellifera* | 3.00E-16 |
|  |  | DGAT2 | EFA83646.1 | *Polysphondylium pallidum* | 1.00E-13 |
| Unigene37843 | 700 | MGAT2 | XP_001517209.1 | *Ornithorhynchus anatinus* | 2.00E-27 |
|  |  | DGAT2 | XP_002708732.1 | *Oryctolagus cuniculus* | 2.00E-24 |
| Unigene5365 | 511 | MGAT1 | XP_002712545.1 | *Oryctolagus cuniculus* | 6.00E-09 |
|  |  | DGAT2 | XP_001495352.2 | *Equus caballus* | 3.00E-06 |
| Unigene10239 | 346 | MGAT2 | XP_975117.1 | *Tribolium castaneum* | 1.00E-13 |
|  |  | DGAT | XP_001602288.1 | *Nasonia vitripennis* | 1.00E-12 |
| Unigene21299 | 235 | MGAT2 | XP_975146.1 | *Tribolium castaneum* | 2.00E-13 |
|  |  | DGAT2 | XP_635762.1 | *Dictyostelium discoideum* | 1.00E-11 |
| Unigene33773 | 451 | MGAT3 | XP_002722873.1 | *Oryctolagus cuniculus* | 4.00E-16 |
|  |  | DGAT2 | NP_001025367.1 | *Danio rerio* | 2.00E-14 |
| Unigene18121 | 212 | MGAT3 | XP_001154107.1 | *Pan troglodytes* | 5.00E-07 |
|  |  | DGAT2 | XP_001083431.1 | *Macaca mulatta* | 1.00E-08 |
| Unigene39897 | 986 | MGAT3 | XP_850305.1 | *Canis familiaris* | 5.00E-43 |
|  |  | DGAT2 | XP_001602288.1 | *Nasonia vitripennis* | 4.00E-46 |
| Unigene26603 | 284 | MGAT2 | XP_393396.2 | *Apis mellifera* | 6.00E-17 |
|  |  | DGAT2 | XP_001653515.1 | *Aedes aegypti* | 3.00E-17 |
| Unigene10595 | 533 | DGAT | AAL78365.1 | *Drosophila melanogaster* | 6.00E-34 |
| Unigene18654 | 216 | DGAT1 | AAL78365.1 | *Drosophila melanogaster* | 1.00E-18 |
| Unigene2733 | 201 | DGAT | AAL78365.1 | *Drosophila melanogaster* | 3.00E-12 |
| Unigene37484 | 668 | DGAT | AAL78365.1 | *Drosophila melanogaster* | 1.00E-54 |
| Unigene22969 | 247 | ACAT | XP_001663352.1 | *Aedes aegypti* | 2.00E-07 |
| Unigene40687 | 1216 | ACAT | XP_001951433.1 | *Acyrthosiphon pisum* | 1.00E-103 |
