# Supplementary material for: Transcriptome Analysis of the Chinese White Wax Scale Ericerus pela with Focus on Genes Involved in Wax Biosynthesis
Source: PLoS One. 2012 Apr 20;7(4):e35719. doi: 10.1371/journal.pone.0035719 (PMC3334986; doi:10.1371/journal.pone.0035719)
Supplement: Table S4 — ABC transporter genes identified in the protein database. (DOC) [file pone.0035719.s004.doc]

**Table S4** ABC transporter genes identified in the protein database.

| Unigene ID | Length | Protein database | Subject ID | Species | E value |
| --- | --- | --- | --- | --- | --- |
| Unigene10260 | 385 | NR | XP_001650571.1 | *Aedes aegypti* | 4.00E-18 |
| Unigene13178 | 1095 | Swissprot | XP_001945365.1 | *Acyrthosiphon pisum* | 1.00E-72 |
| Unigene14233 | 733 | KEGG | K05663 |  | 2.00E-65 |
| Unigene15426 | 601 | NR | XP_001945365.1 | *Acyrthosiphon pisum* | 3.00E-20 |
| Unigene15433 | 1104 | Swissprot | Q8T674 | *Dictyostelium discoideum* | 4.00E-39 |
| Unigene17604 | 209 | NR | XP_001653357.1 | *Aedes aegypti* | 6.00E-09 |
| Unigene20320 | 228 | Swissprot | Q9C8H0 | *Arabidopsis thaliana* | 9.00E-22 |
| Unigene23023 | 248 | COG | COG0488 |  | 2.00E-34 |
| Unigene23396 | 250 | Swissprot | Q9C8H0 | *Arabidopsis thaliana* | 5.00E-25 |
| Unigene23553 | 252 | NR | AAL91490.1 | *Dictyostelium discoideum* | 1.00E-06 |
| Unigene2506 | 981 | Swissprot | Q8T674 | *Dictyostelium discoideum* | 7.00E-42 |
| Unigene25393 | 270 | NR | XP_001945365.1 | *Acyrthosiphon pisum* | 3.00E-11 |
| Unigene29841 | 338 | NR | XP_001945365.1 | *Acyrthosiphon pisum* | 2.00E-12 |
| Unigene29969 | 341 | Swissprot | Q9M1Q9 | *Arabidopsis thaliana* | 8.00E-10 |
| Unigene309 | 481 | Swissprot | Q8T674 | *Dictyostelium discoideum* | 7.00E-15 |
| Unigene31274 | 372 | NR | EFA81848.1 | *Polysphondylium pallidum* | 4.00E-41 |
| Unigene31421 | 376 | NR | XP_973493.1 | *Tribolium castaneum* | 2.00E-36 |
| Unigene3230 | 202 | NR | XP_638739.1 | *Dictyostelium discoideum* | 3.00E-07 |
| Unigene32596 | 409 | Swissprot | Q9MAH4 | *Arabidopsis thaliana* | 7.00E-07 |
| Unigene33779 | 451 | NR | XP_001605354.1 | *Nasonia vitripennis* | 4.00E-41 |
| Unigene33884 | 456 | KEGG | K05663 |  | 2.00E-54 |
| Unigene34848 | 498 | Swissprot | Q54U44 | *Dictyostelium discoideum* | 2.00E-15 |
| Unigene35666 | 541 | Swissprot | Q8T674 | *Dictyostelium discoideum* | 3.00E-32 |
| Unigene35830 | 552 | KEGG | K05663 |  | 1.00E-67 |
| Unigene36570 | 597 | NR | XP_001327851.1 | *Trichomonas vaginalis* | 2.00E-09 |
| Unigene37951 | 712 | NR | XP_001868541.1 | *Culex quinquefasciatus* | 2.00E-66 |
| Unigene37983 | 715 | Swissprot | Q8T674 | *Dictyostelium discoideum* | 2.00E-06 |
| Unigene38542 | 774 | NR | XP_001945365.1 | *Acyrthosiphon pisum* | 2.00E-59 |
| Unigene40850 | 1293 | NR | XP_001951744.1 | *Acyrthosiphon pisum* | 0 |
| Unigene41117 | 1432 | NR | XP_974441.2 | *Tribolium castaneum* | 0 |
| Unigene41125 | 1437 | Swissprot | Q8T674 | *Dictyostelium discoideum* | 6.00E-28 |
| Unigene41206 | 1495 | COG | COG0488 |  | 1.00E-150 |
| Unigene41591 | 1942 | COG | COG0488 |  | 1.00E-158 |
| Unigene41623 | 2005 | NR | XP_001950956.1 | *Acyrthosiphon pisum* | 0 |
| Unigene41642 | 2042 | COG | COG0488 |  | 1.00E-06 |
| Unigene41747 | 2411 | Swissprot | Q8T674 | *Dictyostelium discoideum* | 1.00E-75 |
| Unigene41814 | 3303 | COG | COG0488 |  | 1.00E-111 |
| Unigene4551 | 751 | NR | XP_001653363.1 | *Aedes aegypti* | 6.00E-39 |
| Unigene503 | 754 | Swissprot | Q9FNB5 | *Arabidopsis thaliana* | 3.00E-10 |
| Unigene5337 | 852 | Swissprot | Q8T674 | *Dictyostelium discoideum* | 2.00E-16 |
| Unigene5526 | 1460 | Swissprot | Q8T674 | *Dictyostelium discoideum* | 4.00E-44 |
| Unigene7734 | 537 | NR | XP_001945365.1 | *Acyrthosiphon pisum* | 2.00E-17 |
| Unigene9338 | 545 | NR | XP_001605354.1 | *Nasonia vitripennis* | 5.00E-09 |
| Unigene9852 | 474 | NR | XP_001653360.1 | *Aedes aegypti* | 6.00E-33 |
