# Supplementary material for: Transcriptome Analysis of the Chinese White Wax Scale Ericerus pela with Focus on Genes Involved in Wax Biosynthesis
Source: PLoS One. 2012 Apr 20;7(4):e35719. doi: 10.1371/journal.pone.0035719 (PMC3334986; doi:10.1371/journal.pone.0035719)
Supplement: Table S5 — Primers used for qRT-PCR. (DOC) [file pone.0035719.s005.doc]

**Table S5** Primers used for qRT-PCR.

| Primer name | Primer sequence (5’-3’) |
| --- | --- |
| ELO1 F | CAGTCAGTCGCTCCAAGTTAT |
| ELO1 R | ATAAAATCCGCTCGTCCAGT |
| ELO2 F | CGTAAACCAACGGGCTGAGG |
| ELO2 R | TCCTGTTCGTCCTGTCCAAGTT |
| FAR1 F | CAGGCGATTGCGAGAAACG |
| FAR1 R | GAATGTGCCAGATCCAGAAGAT |
| FAR2 F | CGATACTTATGCCCGCATGGTT |
| FAR2 R | GTCATCAAATTTCCACTGTTTGGT |
| FAR3 F | TGTTTCACAATGCTGCCTTACT |
| FAR3 R | TTTCTTCCACTTCCGCTTCGT |
| FAR4 F | GTTGGCCTTATGAGGGTCTTG |
| FAR4 R | CCATTTCAGGTACTCGGTCTCT |
| FAR5 F | CAAATGAGCCGATAGCAGGAT |
| FAR5 R | GCATGAACACTTCGCAACAAC |
| ACT1 F | ACCACTCGTACTACCCACAAC |
| ACT1 R | TTGAGGATAAAGTGAGCGAAGT |
| WS1 F | CATCCTTCACTTGGGCATAT |
| WS1 R | CCCATGTGCGGAGTGTTT |
| pWS 1F | GTTACCTGCCCGCAGCCACT |
| pWS1 R | CTTCTCACATCCACATGCGT |
| pWS2 F | GATTACCACGAACCAATGTCC |
| pWS2 R | GCTGCTACTCTGTCGGCGAAT |
| pWS3 F | GGACGACCGAAACCTATTGT |
| pWS3 R | TTCCAGCATCCTTGTACGAC |
| pWS4 F | TCCACCTTCTTCAAGTTCTACCT |
| pWS4 R | TTGACGCTCCTTTGCGATACG |
| pWS5 F | GTATCTGATGGGAAGTTGGC |
| pWS5 R | ACATCTGTCTCGGTTCATTC |
| ABC1 F | GACAAGCGCATACAATTGGTCTAAT |
| ABC1 R | CTTCCTCTAAAGACTGGCAACT |
| Actin F | GTGACGACGATGTTGCTGCTTTG |
| Actin R | CCCATGCCCACCATAATACCCTGAT |
