# Supplementary figures and images for: Transcriptome Analysis of the Chinese White Wax Scale Ericerus pela with Focus on Genes Involved in Wax Biosynthesis
Source: PLoS One. 2012 Apr 20;7(4):e35719. doi: 10.1371/journal.pone.0035719 (PMC3334986; doi:10.1371/journal.pone.0035719)

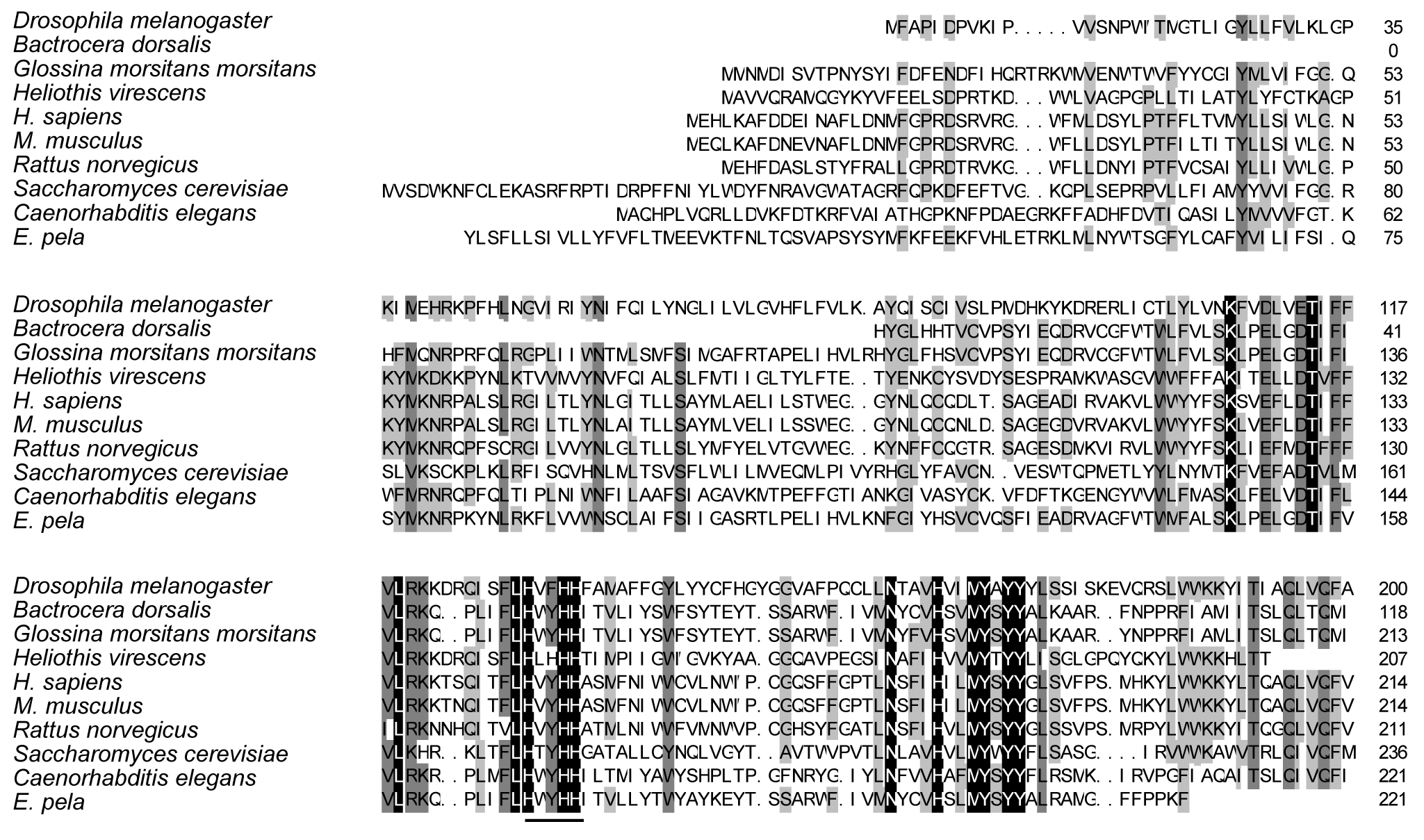

Supplement: Figure S1 — Alignment of the deduced amino acid sequences of ELO. Identical amino acid residues and conservative substitutions are shaded in black or gray respectively. The conserved Histidine box is underlined. The GenBank accession numbers of the sequences are as follows: Drosophila melanogaster NM_141699.3, Bactrocera dorsalis HQ148712.1, Glossina morsitans morsitans ADD19917.1, Heliothis virescens ACX53823.1, H. sapiens NP_060240, M. musculus NP_062296, Rattus norvegicus NP_599209, Saccharomyces cerevisiae NP_012339, Caenorhabditis elegans AF244356. (TIF) [file pone.0035719.s006.tif]

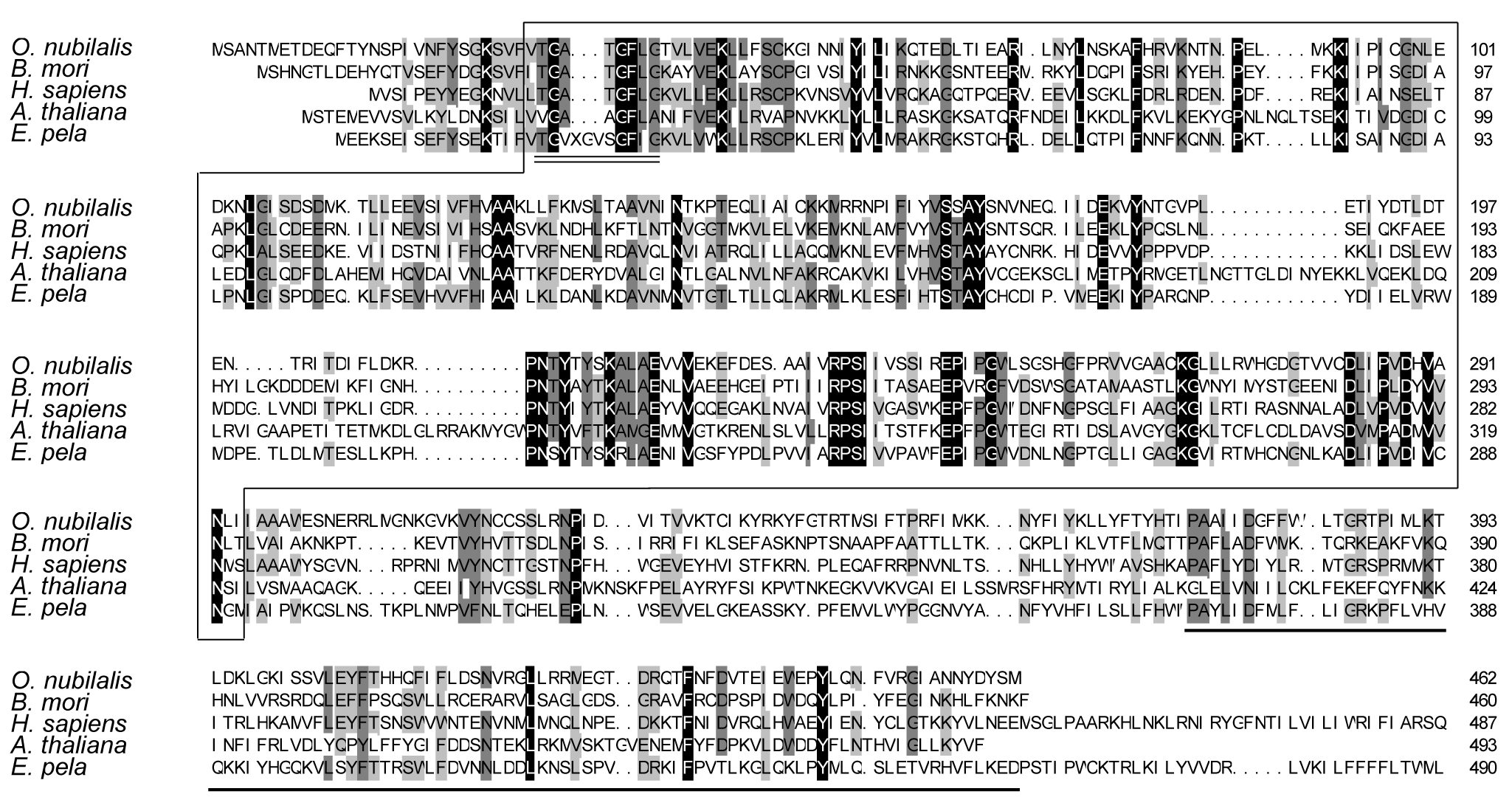

Supplement: Figure S2 — Alignment of the deduced amino acid sequences of FAR. The Rossmann-fold domain is shown in black box, the NADH-binding motif is double underlined, and the Sterile protein domain is underlined. The GenBank accession numbers of the sequences are as follows: O. nubilalis FJ807735, B. mori BAC79426, H. sapiens AAT42129, A. thaliana NP567936. (TIF) [file pone.0035719.s007.tif]

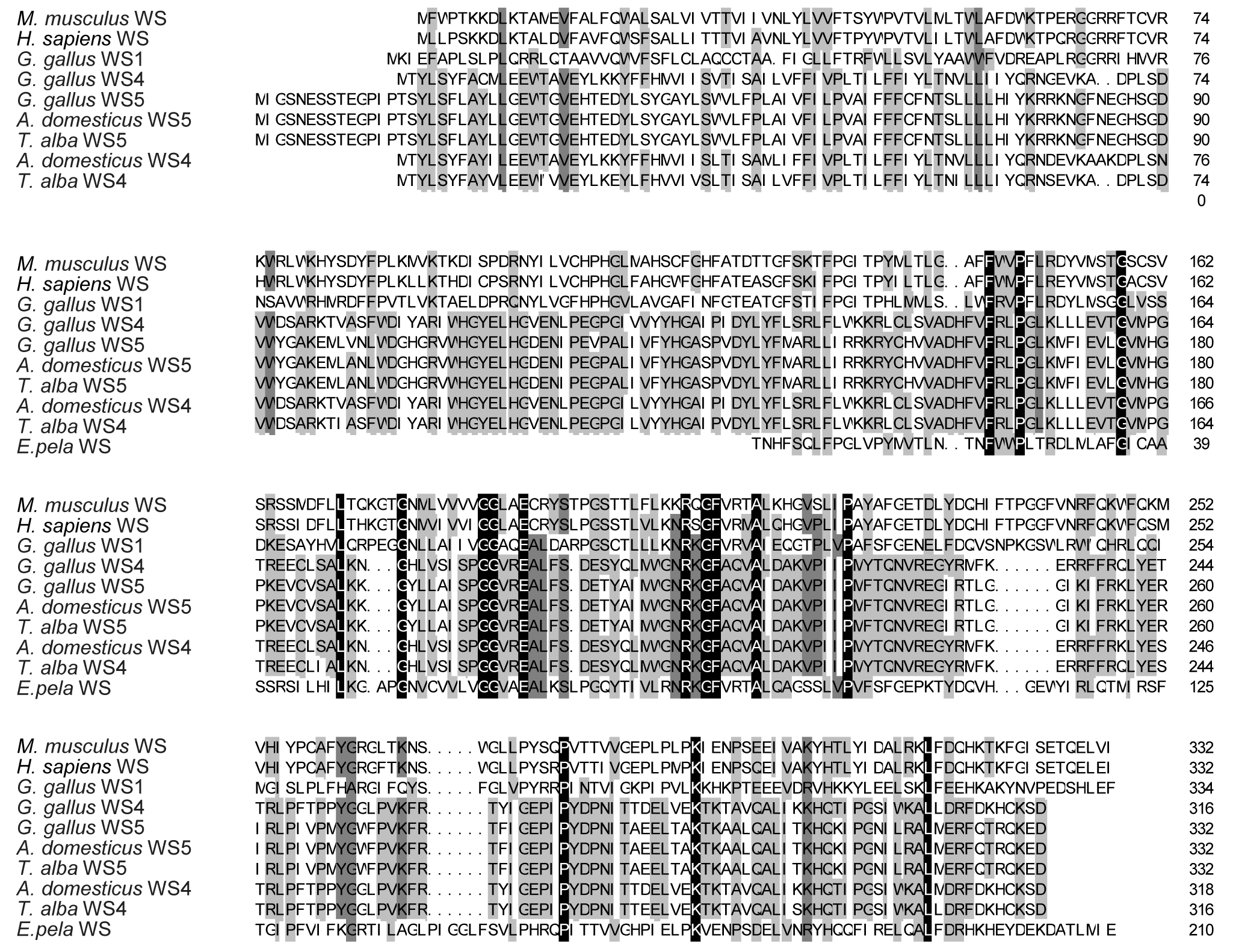

Supplement: Figure S3 — Alignment of the deduced amino acid sequences of WS. The GenBank accession numbers of the sequences are as follows: M. musculus AY611032, H. sapiens AY605053, G. gallus WS1 XP_424082.2, G. gallus WS4 XP_419207.1, G. gallus WS5 NP_001026192.1, A. domesticus WS5 Q031647, T. alba WS5 JQ031646, A. domesticus WS4 JQ031643, T. alba WS4 JQ031645. (TIF) [file pone.0035719.s008.tif]

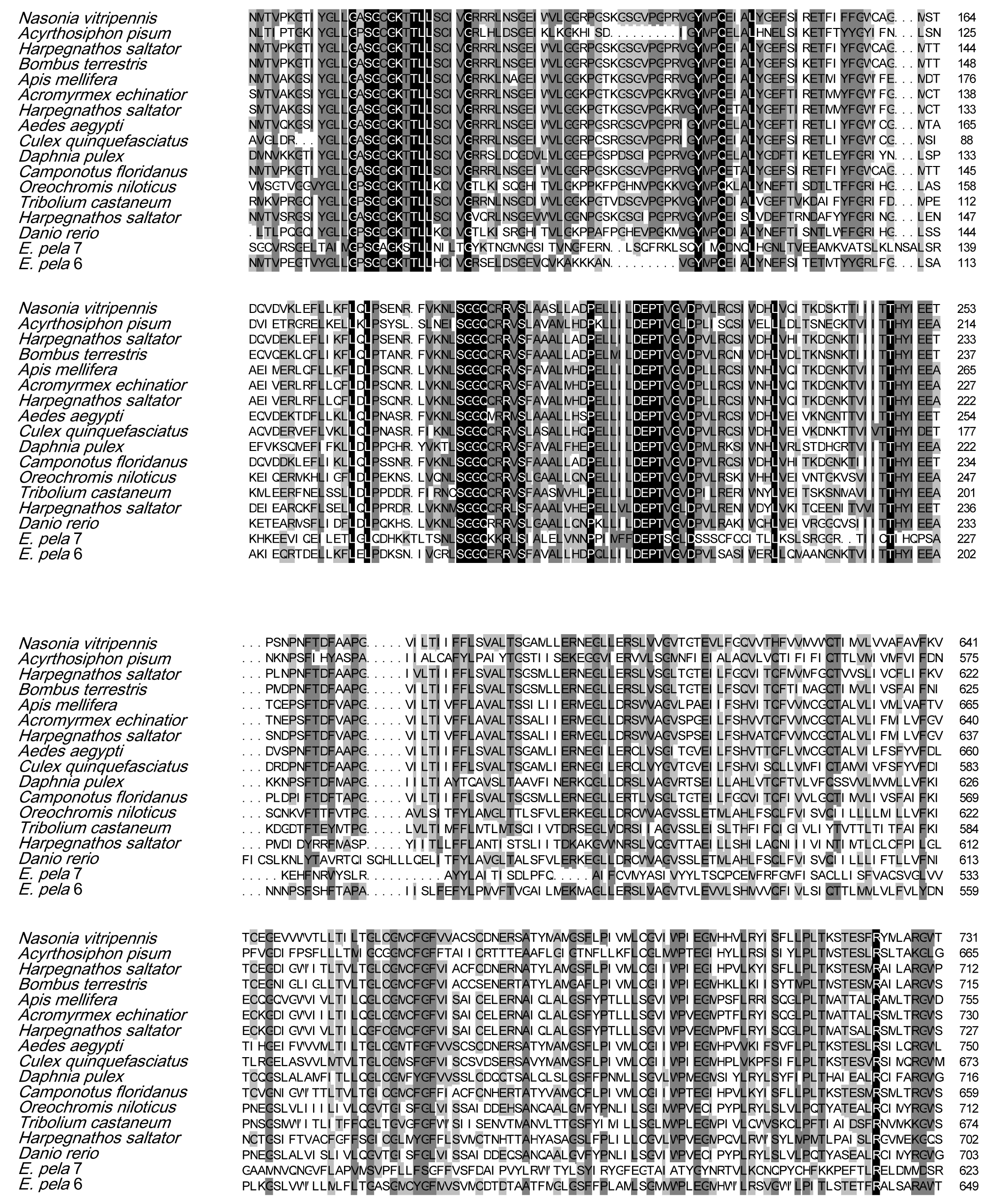

Supplement: Figure S4 — Alignment of the deduced amino acid sequences of ABC transporters. The GenBank accession numbers of the sequences are as follows: Nasonia vitripennis XP_003426604.1, Acyrthosiphon pisum XP_001945365.2, Harpegnathos saltator EFN84917.1, Bombus terrestris XP_003401420.1, Apis mellifera XP_393164.4, Acromyrmex echinatior EGI67545.1, Harpegnathos saltator EFN78194.1, Aedes aegypti XP_001650952.1, Culex quinquefasciatus XP_001862847.1, Daphnia pulex EFX71377.1, Camponotus floridanus EFN69284.1, Oreochromis niloticus XP_003459375.1, Tribolium castaneum XP_973444.1, Harpegnathos saltator EFN84918.1, Danio rerio XP_687003.3. (TIF) [file pone.0035719.s009.tif]

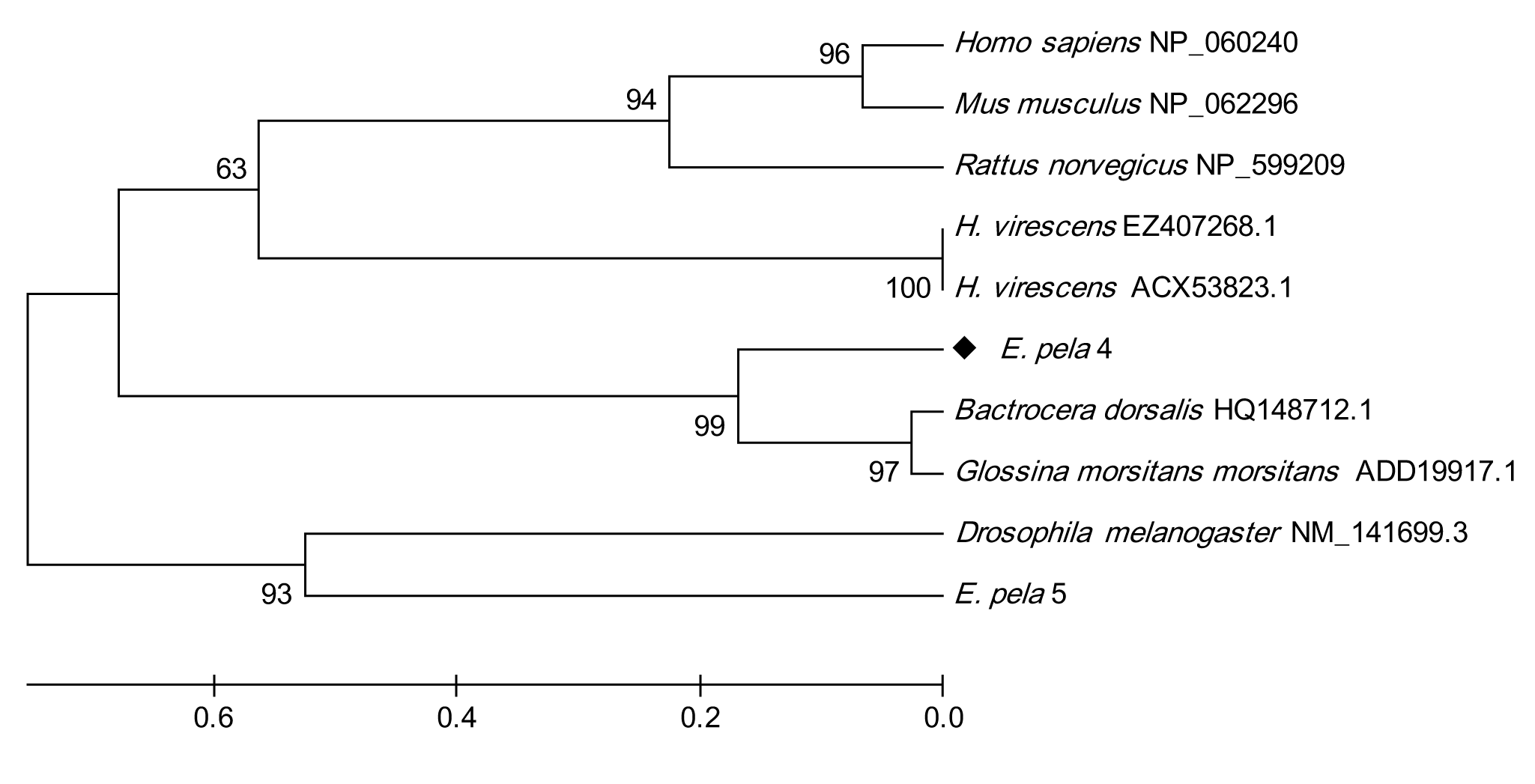

Supplement: Figure S5 — Phylogenetic tree of ELOs. (TIF) [file pone.0035719.s010.tif]

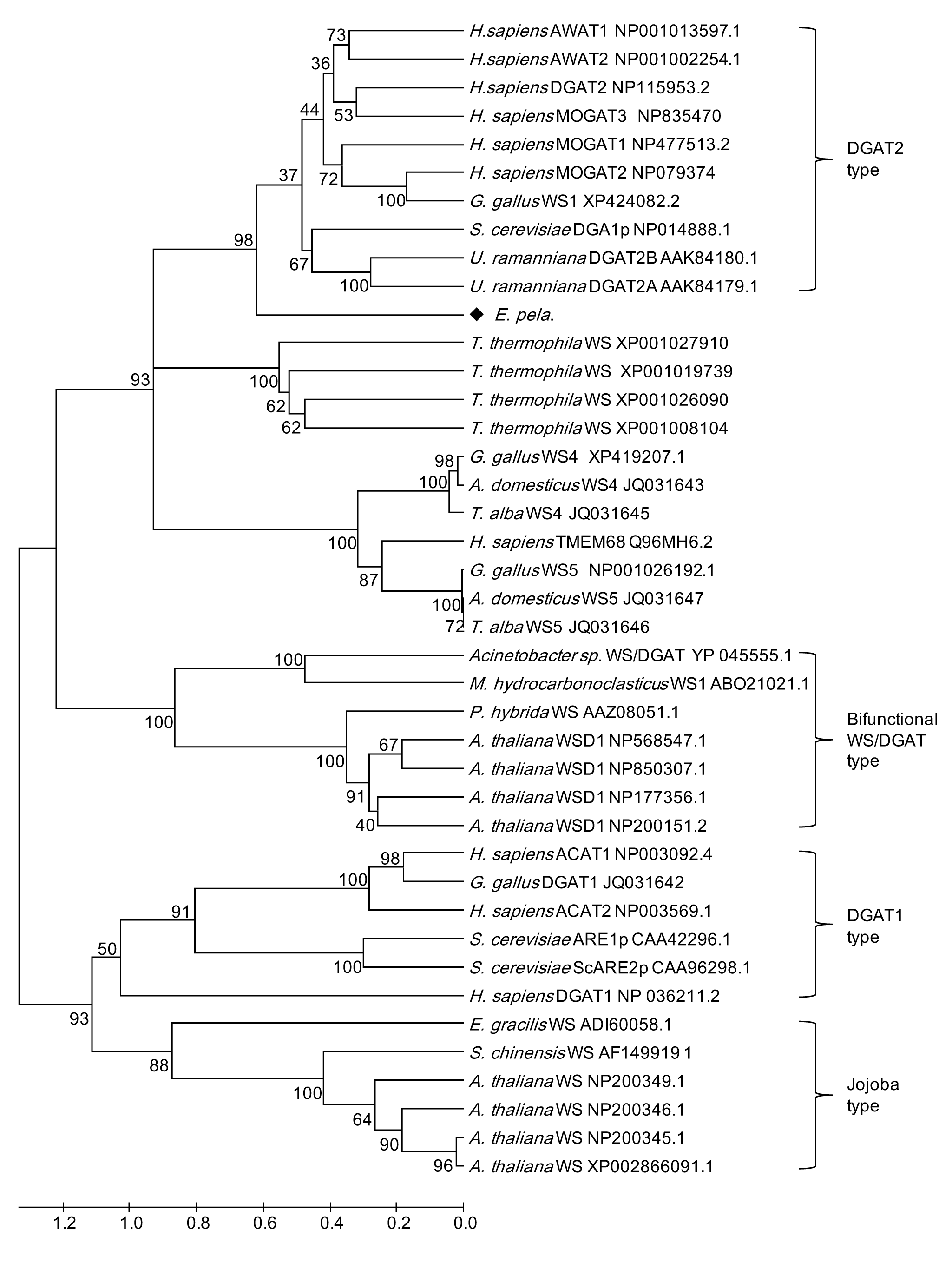

Supplement: Figure S7 — Phylogenetic tree of WSs, DGATs, MOGATs, and ACATs. (TIF) [file pone.0035719.s012.tif]

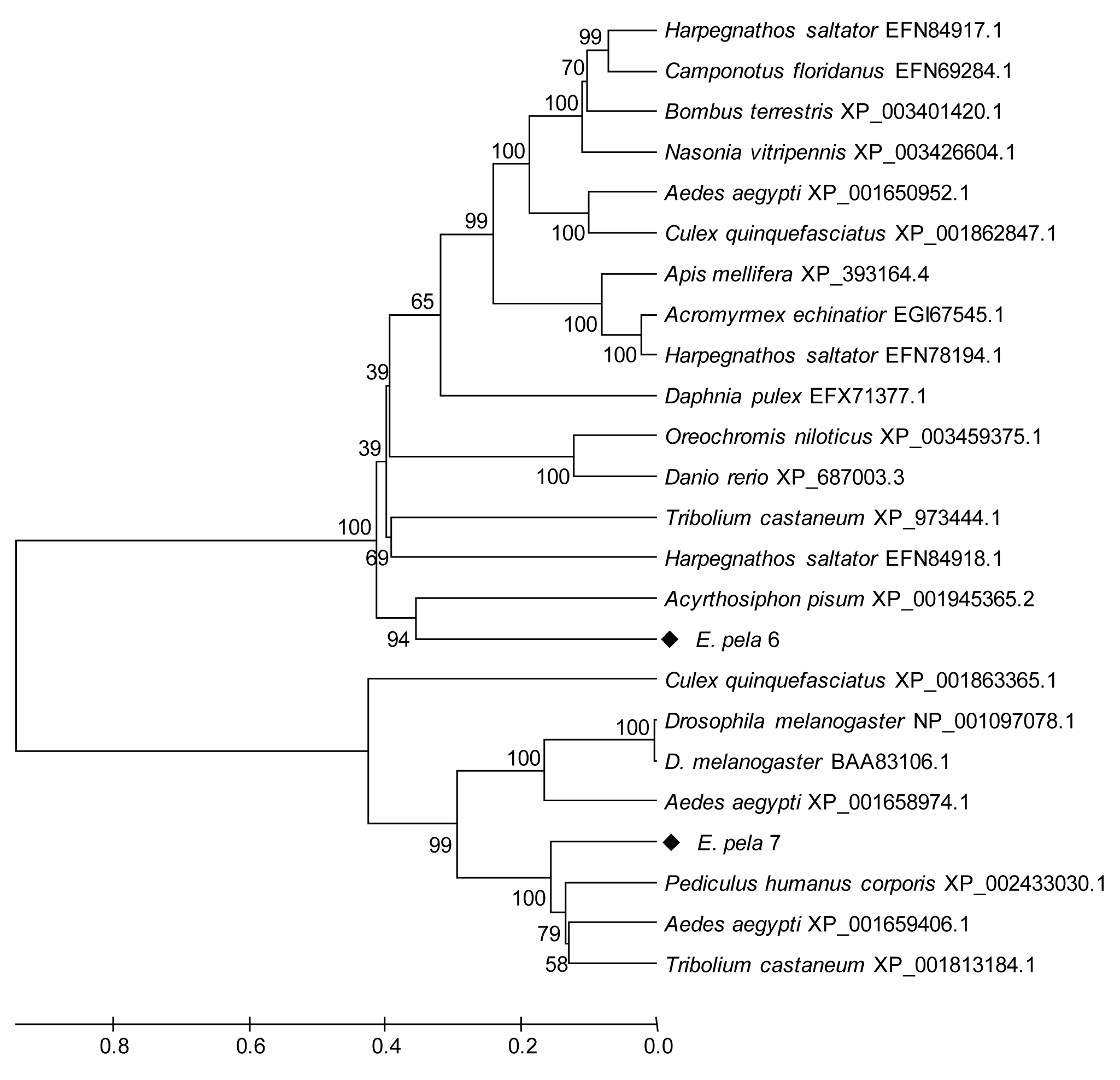

Supplement: Figure S8 — Phylogenetic tree of ABC transporters. (TIF) [file pone.0035719.s013.tif]
